# Patient Outcomes in Disorders of Consciousness Following Transcranial Magnetic Stimulation: A Systematic Review and Meta-Analysis of Individual Patient Data

**DOI:** 10.3389/fneur.2021.694970

**Published:** 2021-08-12

**Authors:** Christen M. O'Neal, Lindsey N. Schroeder, Allison A. Wells, Sixia Chen, Tressie M. Stephens, Chad A. Glenn, Andrew K. Conner

**Affiliations:** ^1^Department of Neurosurgery, University of Oklahoma Health Sciences Center, Oklahoma City, OK, United States; ^2^Biostatistics and Epidemiology, University of Oklahoma Health Sciences Center, Oklahoma City, OK, United States

**Keywords:** transcranial magnetic stimulation, minimally conscious state, persistent vegetative state, disorders of consciousness, individual patient data

## Abstract

**Background:** There are few treatments with limited efficacy for patients with disorders of consciousness (DoC), such as minimally conscious and persistent vegetative state (MCS and PVS).

**Objective:** In this meta-analysis of individual patient data (IPD), we examine studies utilizing transcranial magnetic stimulation (TMS) as a treatment in DoC to determine patient and protocol-specific factors associated with improved outcomes.

**Methods:** We conducted a systematic review of PubMed, Ovid Medline, and Clinicaltrials.gov through April 2020 using the following terms: “minimally conscious state,” or “persistent vegetative state,” or “unresponsive wakefulness syndrome,” or “disorders of consciousness” and “transcranial magnetic stimulation.” Studies utilizing TMS as an intervention and reporting individual pre- and post-TMS Coma Recovery Scale-Revised (CRS-R) scores and subscores were included. Studies utilizing diagnostic TMS were excluded. We performed a meta-analysis at two time points to generate a pooled estimate for absolute change in CRS-R Index, and performed a second meta-analysis to determine the treatment effect of TMS using data from sham-controlled crossover studies. A linear regression model was also created using significant predictors of absolute CRS-R index change.

**Results:** The search yielded 118 papers, of which 10 papers with 90 patients were included. Patients demonstrated a mean pooled absolute change in CRS-R Index of 2.74 (95% CI, 0.62–4.85) after one session of TMS and 5.88 (95% CI, 3.68–8.07) at last post-TMS CRS-R assessment. The standardized mean difference between real rTMS and sham was 2.82 (95% CI, −1.50 to 7.14), favoring rTMS. The linear regression model showed that patients had significantly greater CRS-R index changes if they were in MCS, had an etiology of stroke or intracranial hemorrhage, received 10 or more sessions of TMS, or if TMS was initiated within 3 months from injury.

**Conclusions:** TMS may improve outcomes in MCS and PVS. Further evaluation with randomized, clinical trials is necessary to determine its efficacy in this patient population.

## Introduction

Recovery from minimally conscious and persistent vegetative states, MCS and PVS, respectively, can vary widely depending on time from injury and etiology. A recent meta-analysis of natural history in disorders of consciousness (DoC) demonstrated emergence from PVS to MCS in patients with traumatic injuries had a change in diagnosis from PVS to MCS at a rate of 38% at 3 months and 67% at 6 months (1). However, improvement from PVS to MCS in patients with non-traumatic injuries ranged from 7.5 to 17%, depending on DoC status at 6 months post-injury, suggesting anoxia and stroke may portend a poor prognosis (1). Although MCS recovery rates are often described as being more favorable than for PVS, also known as unresponsive wakefulness syndrome (UWS), this evidence is limited. Natural history studies have not yet elucidated the average rate of recovery for patients in DoC, let alone defined these rates for each etiology (1–3).

There are few interventions that have demonstrated any level of improvement in DoC patients. Many of the interventions that have been explored target dopaminergic pathways, which have been implicated in consciousness and disorders affecting consciousness (4). Currently amantadine, an NMDA antagonist and dopamine agonist that upregulates postsynaptic dopamine receptors to increase available dopamine (5), is the only treatment recommended by the American Academy of Neurology for DoC (2). A randomized, controlled trial of amantadine as a treatment for patients with MCS or PVS due to acute traumatic brain injury (TBI) demonstrated increased rates of disability rating scale (DRS) score changes in those receiving amantadine compared to placebo during the treatment phase of the study (6). However, patients in the treatment group demonstrated less change in DRS scores compared to placebo once amantadine was discontinued and the overall effect of amantadine was not significant at the conclusion of the study (6). More recent studies have explored repetitive transcranial magnetic stimulation (rTMS) as a possible intervention in DoC. TMS works by inducing a focal magnetic field with a coil over the scalp, thereby generating intracranial currents (7, 8). While amantadine works through global upregulation of dopaminergic pathways, rTMS is hypothesized to work via a more targeted mechanism based on the site of stimulation (7). The dorsolateral prefrontal cortex (DLPFC), a key component of the central executive network (CEN), is often targeted in patients with DoC with the goal of repairing disrupted balance of activation between the CEN and default mode network (DMN) (9). Stimulation of DLPFC facilitates dopaminergic-driven connectivity, which modulates internetwork connectivity between CEN and DMN via the salience network (7, 9–11). Despite the potential of rTMS to facilitate changes in connectivity, there is a clear absence of large-scale, randomized-controlled trials evaluating TMS as an intervention for DoC patients, with the largest randomized trials involving 20 patients (12, 13). Transcranial direct current stimulation (tDCS) has also been studied in patients with DoC and demonstrated positive results in several studies, which have been recently reviewed (14). Similar to rTMS, tDCS has been shown to facilitate changes in internetwork connectivity [see (15) for a more comprehensive review of the mechanism of tDCS].

In 2018, the American Academy of Neurology updated the practice guidelines concerning treatment of patients in MCS or PVS (2). While the guidelines provided the highest level of evidence available based on a thorough systematic review with a rigorous methodology, studies utilizing TMS did not meet the inclusion criteria (3). For this reason, TMS as an intervention in patients with DoC was not examined in the systematic review or the guidelines (2, 3), a point that drew criticism from others in the field (16). Rather, the guidelines recommended the use of amantadine as the only intervention with probable efficacy and cautioned clinicians to counsel patients' families on the potential dangers of other interventions that have not been fully evaluated (2). Nevertheless, Whyte and Nakase-Richardson suggest it may be impractical to wait for stronger evidence due to the high costs associated with inpatient care for DoC patients, which would be necessary for any experimental study in this patient population (17). Instead, they emphasize utilizing the best evidence currently available (17).

Meta-analyses and systematic reviews of randomized-controlled trials are the highest level of evidence available among clinical studies, though this methodology may not be feasible for all patient populations or disease processes. Collecting and analyzing individual patient data (IPD) using a systematic methodology is one way to overcome limited studies with smaller patient cohorts (18). In this meta-analysis of IPD, we examine studies utilizing TMS interventions in DoC and the effect of multiple patient and protocol-specific factors with respect to patient outcomes.

## Materials and Methods

### Search Strategy and Eligibility Criteria

Preferred Reporting Items for Systematic Reviews and Meta-Analyses (PRISMA) guidelines were utilized to conduct a systematic review of studies utilizing TMS as an intervention for MCS or PVS. A search of PubMed, Ovid Medline, and ClinicalTrials.gov was conducted through April 5th, 2020 using the following terms: “minimally conscious state,” or “persistent vegetative state,” or “unresponsive wakefulness syndrome,” or “disorders of consciousness” and “transcranial magnetic stimulation.” The following inclusion criteria were utilized: (1) papers reported individual pre- and post-TMS Coma Recovery Scale-Revised (CRS-R) scores, (2) included a patient diagnosed with MCS, PVS, or UWS, and (3) TMS was utilized as an intervention for a patient diagnosed with a disorder of consciousness. Studies focusing on diagnostic or prognostic applications of TMS, including those utilizing a single-pulse protocol, were excluded. Additionally, the authors only included research that was original, and excluded any narrative or systematic reviews or meta-analyses. Articles that were not accessible in English were also excluded. Inter-rater reliability for papers meeting inclusion criteria was assessed using Cohen's Kappa.

### Data Extraction and Outcome Measures

The following study-specific data points were extracted: first author, year of publication, study design, control conditions such as sham TMS, exclusion criteria and number of included patients, type of TMS protocol utilized, including intensity, frequency, number of sessions, stimulation site, and all CRS-R scores and subscores available for each patient. The following patient-specific data points were also extracted: age, sex, etiology of injury, time from injury to TMS intervention, and any reported adverse TMS effects.

The CRS-R scale has been shown to have excellent inter-rater and test-retest reliability (19, 20). This scale has also been shown to be sensitive to differentiating MCS from PVS (19), and has demonstrated prognostic value for improvement in DoC status when examining change from initial score to 4 weeks post-injury (21). However, the CRS-R score is not a linear scale of consciousness and has limited applications in statistical analyses (22). Modifications to the CRS-R score can aid in statistical analysis and may be calculated using CRS-R subscores (22). For this reason, we chose absolute change in CRS-R index as the primary outcome in our analysis (22). In order to convert CRS-R scores to the CRS-R index, subscores were categorized into reflexive and cognitively mediated behaviors (23). Although this categorization has been previously described by Sattin et al. for the CRS-R Modified Score (CRS-R-MS), it does not allow patients to receive points for lower-scored items that they were not assessed on, meaning CRS-R assessments that follow current guidelines are unable to be converted to the CRS-R-MS (22, 23). For this reason, we utilized the CRS-R index, which complies with current guidelines for administering the CRS-R assessment by assuming patients will receive points for all lower-scored items within a domain, without requiring patients to undergo testing for all items of the specified domain (22). Then, the sum of all reflexive behavior scores for a patient at a given time point were divided by the maximum reflexive behavior score of 7, to create the reflex behavior index (22). The same procedure was followed for cognitively mediated behaviors to create the cognitively mediated behavior index, though the maximum cognitively mediated behavior score of 11 was used (22). The two calculated indices were then utilized to find the transposition matrix value, as previously described (22). The arousal domain of the CRS-R score was then divided by 3 to give the arousal scale (22). Lastly, the transposition matrix value was added to the arousal scale (22). The final calculated value was the CRS-R Index.

To account for differences between the transient effects of TMS immediately following one session of TMS and the long-term effects of TMS, CRS-R subscores were collected and converted to the CRS-R index at the following time points: (1) baseline score prior to TMS, (2) score after one session of TMS, and (3) the last TMS score recorded for the patient. The difference between the baseline score and either endpoint was used to calculate the absolute change in CRS-R Index so that two separate analyses of the immediate and longer-term effects of TMS were conducted as follows:

(1) Absolute change in CRS-R Index After One Session = (CRS-R Index Score After One Session of TMS) – (Baseline CRS-R Index Score)
(2) Absolute change in CRS-R Index at Last Post-TMS CRS-R Assessment = (CRS-R Index Score at Last Post-TMS CRS-R Assessment) – (Baseline CRS-R Index Score)

Response to TMS, defined as any change in CRS-R score above a patient's baseline pre-TMS CRS-R score, was included as a secondary outcome. This secondary outcome was included to make qualitative comparisons across multiple patient and protocol-specific factors. These qualitative comparisons were descriptive in nature, as opposed to a true statistical analysis of the effect these variables had on patient response, and are therefore supplementary to the data provided in our later analysis of absolute change in CRS-R Index using a linear regression model.

Both reviewers checked IPD for all patients. Any suspected duplicate patients were discussed and agreed upon by both reviewers prior to exclusion. Additionally, any patients with a baseline CRS-R score of 23 were excluded.

### Handling of Missing Data and Categorization of Variables

Sex and exact age were not provided by all studies. Liu et al. provided 5-year ranges for patient ages and therefore we used the midpoint of the age range to calculate the approximate age for each patient (24). Sex was also not reported in Liu et al. and therefore these patients were excluded from the analysis of sex as a predictor in the linear regression model (24). The continuous data for time from injury to TMS was dichotomized by patients who received TMS at three or more months post-injury vs. prior to 3 months. Stimulation site was also grouped by studies stimulating M1 vs. studies stimulating cortical areas that lead to modification of DMN connectivity, such as DLPFC, angular gyrus, and inferior parietal lobe, which we have grouped as non-M1 (7, 25).

### Risk of Bias and Quality Assessment

The Grading of Recommendations, Assessment, Development, and Evaluation (GRADE) guidelines were followed to assign an evidence profile for the outcome of interest using the GRADEpro Guideline Development Tool (26–28). Risk of bias was assessed using ROBINS (29).

### Individual Study Effect Estimates

Patient outcomes were measured using CRS-R scores and subscores, which were converted to CRS-R Index measures at all time points for which data was available. Absolute change was estimated for each patient by calculating the difference in score from baseline to the following two end-points: (1) assessment after one session of TMS and (2) the last post-TMS CRS-R assessment. Then, each patients' absolute change was used to calculate the pooled estimate for the standardized mean difference and confidence intervals for each study at both end-points.

As a secondary analysis, the treatment effect was evaluated for patients in the last post-TMS CRS-R assessment group if they had sham TMS data available. Absolute change in CRS-R Index was calculated for each included patient for the sham and real r-TMS conditions. Then, the difference between sham and real rTMS absolute change was used to calculate treatment effect for each patient as follows:

(1) Absolute change in CRS-R Index at Last Post-Sham CRS-R Assessment = (CRS-R Index Score at Last Post-Sham CRS-R Assessment) – (Baseline CRS-R Index Score)
(2) Absolute change in CRS-R Index at Last Post-TMS CRS-R Assessment = (CRS-R Index Score at Last Post-TMS CRS-R Assessment) – (Baseline CRS-R Index Score)
(3) Treatment Effect = (Absolute change in CRS-R Index at Last Post-TMS CRS-R Assessment) – (Absolute change in CRS-R Index at Last Post-Sham CRS-R Assessment)

Lastly, the individual treatment effects were pooled to calculate an overall treatment effect for each study. Due to limited studies with available control data, only three studies were included, with a total of 14 patients analyzed.

### Summary Effect Estimates

A one-stage random-effects meta-analysis of IPD was conducted using R version 3.5.2 “meta” package to generate the pooled mean absolute change in CRS-R Index for two conditions: (1) from baseline to assessment after one session of TMS and (2) from baseline to the last post-TMS CRS-R assessment. Then, a second meta-analysis was performed using data from crossover studies with a sham-controlled condition to assess treatment effect. Studies were designated a weight based on sample size and variation. Any studies with a standard deviation of 0 were not given weight in the analysis. Heterogeneity was assessed using Higgins' I statistic. Forest plots were created in GraphPad Prism version 9.0 for Windows using the analysis generated by R version 3.5.2 “meta” package.

### Additional Statistical Analysis: Paired *T*-Tests

Since the sample sizes included in the three meta-analyses were relatively small, we also performed three paired *t*-tests using SAS 9.4 to determine whether mean post-TMS CRS-R Index measures were significantly higher than mean baseline scores. The following post-TMS time points were tested: (1) after one session of TMS, (2) at the last post-TMS score reported, excluding patients with only one post-TMS measurement after one session of TMS, and (3) at the last post-TMS score reported, including all patients. The last paired *t*-test allowed us to pool together all 87 patients included over the three meta-analyses, as well as the three patients reported in case reports, so that a total of 90 patients were included.

### Additional Statistical Analysis: Absolute Change in CRS-R Index Linear Regression Model

Lastly, a univariate analysis was conducted to determine significant predictors of absolute change in CRS-R Index at the last post-TMS CRS-R assessment. We first used a mixed effect model to analyze “study ID” as a random effect for the outcome variable absolute change in CRS-R Index at the last post-TMS CRS-R assessment. We analyzed the following variables as predictors: number of sessions, frequency, intensity, stimulation site, DoC status, age, sex, time to TMS, and etiology. However, the intra-cluster correlation was <0.05 and the *p*-value for the random effect was not significant, therefore we used a linear regression model instead. We first fit the univariate linear regression model by using the aforementioned individual predictors one at a time. We then fit the multivariate linear regression model for absolute change in CRS-R Index at the last post-TMS CRS-R assessment by including the significant predictors, as determined by the univariate linear regression model. Since Type III analysis of the predictors' two-way interaction terms was not significant, we fit the final model by only including the significant predictors.

## Results

**Figure 1:**
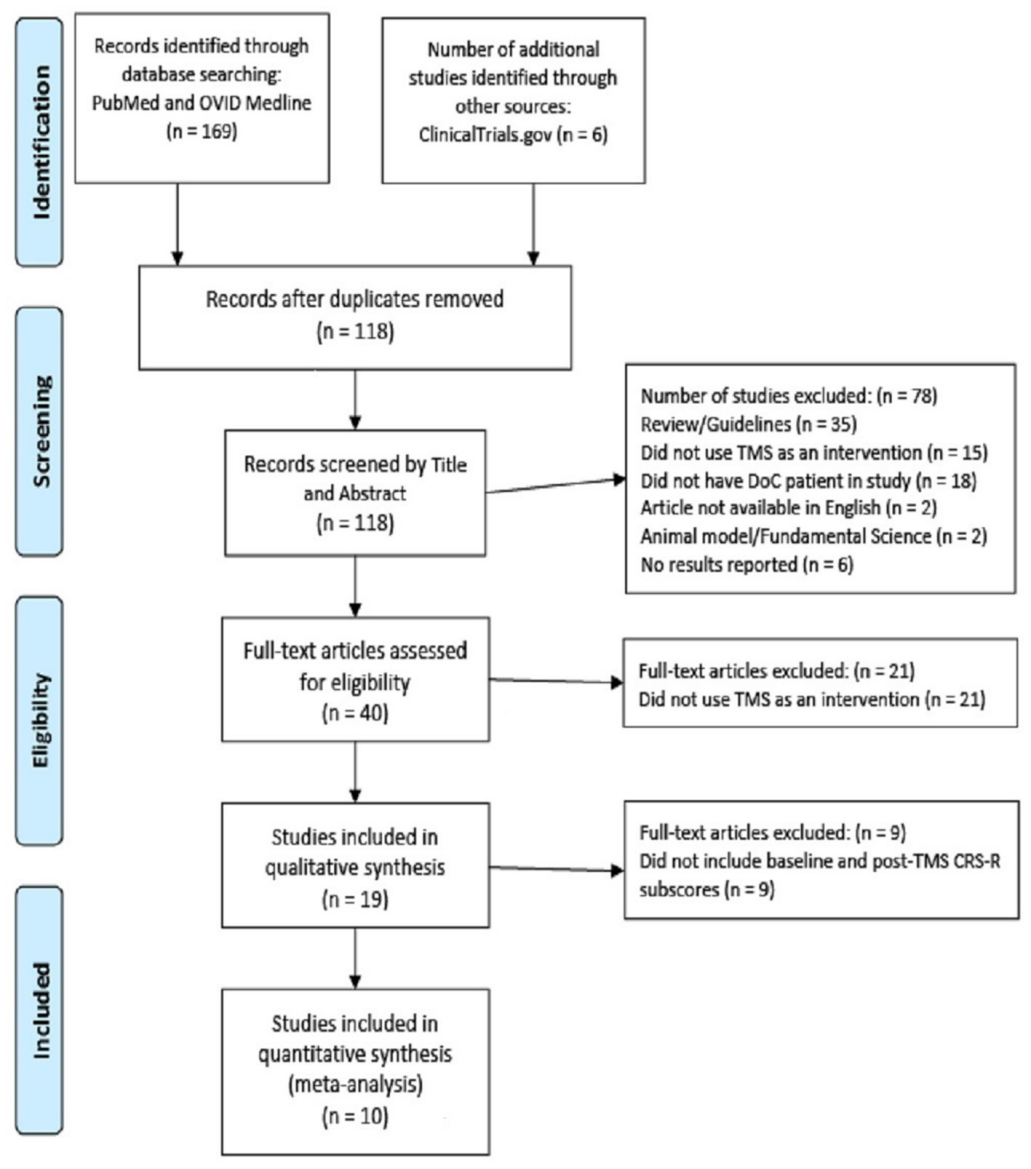
PRISMA diagram. CRS-R, Coma Recovery Scale-Revised; DoC, Disorder of Consciousness; PRISMA, Preferred Reporting Items for Systematic Reviews and Meta-Analyses; TMS, Transcranial magnetic stimulation.

The literature search yielded 175 papers, with 118 studies after duplicates were removed (Figure 1). After screening by abstract and title, 40 studies were accessed as full-text articles. After review, 10 articles with 90 patients were selected for inclusion into the systematic review. Of those studies, three were included in the first synthesis of IPD to determine the pooled mean absolute CRS-R Index change after one session of TMS, five were included in the second synthesis of IPD to determine the pooled mean absolute CRS-R Index change from baseline to last post-TMS CRS-R assessment, and three were included in the final meta-analysis assessing treatment effect of rTMS vs. sham TMS (24, 30, 31). Inter-rater reliability was very good, with κ = 0.907 (95% CI, 0.842–0.972), *p* < 0.0005. No adverse effects to TMS were reported in any studies.

### Study, Patient, and Protocol Characteristics

See Table 1 for an overview of included studies and Supplementary Table 1 for detailed characteristics. See Table 2 for an overview of patient and protocol characteristics and Supplementary Table 2 for IPD by study. Additional characteristics of patients categorized as “responders” can be found in Tables 3, 4. In general, patients with an MCS diagnosis were more likely to respond to TMS than those in PVS (71.4 vs. 22.9% overall) (Table 3). In both MCS and PVS groups, patients with an etiology of stroke/ICH were more likely to respond, with 83.3% of MCS and 45.5% of PVS patients showing response. Table 4 illustrates the number of MCS and PVS patients with improvement by CRS-R subscore domains. Although no PVS patients demonstrated improvement in the oromotor/verbal or arousal CRS-R subscore domains, oromotor/verbal and arousal subscores improved in 8 and 9 MCS patients, respectively. More than half of MCS patients demonstrating any response had improvement in visual subscores (19/30; 63.3%), while PVS patients with any response were most likely to improve in the motor subscore domain (8/11; 72.7%).

**Table 1:** Overview of included studies.

**References**	**Design**	**Patients**		**Etiology**		**rTMS protocol**	**Stimulation target**	**Adverse effects recorded**
Bai et al. (32)	Case report	MCS	1	Stroke/ICH	1	20 sessions, 10 Hz rTMS (90% RMT)	Left DLPFC	None
He et al. (30)	Randomized, sham-controlled crossover study	MCS PVS	1 3	Anoxic	1	5 sessions, 20 Hz rTMS (100% RMT) 5 sessions, sham rTMS with 1-week washout	Left M1	–
				Stroke/ICH	1			
				TBI	2			
Jang et al. (33)	Case report	PVS	1	Stroke/ICH	1	Approximately 121 sessions, 10 Hz rTMS (80% RMT) over 2 months	Right DLPFC	–
Legostaeva et al. (34)	Case series	MCS PVS	22 16	Anoxic	26	10 sessions, 20 Hz rTMS (80% RMT; 2 pts: 40% MO) over 2 weeks	Left angular gyrus	None
				TBI	12			
Lin et al. (35)	Case report	MCS	1	Stroke/ICH	1	14 sessions, 1.5 mA tDCS and 5 Hz rTMS (70% RMT) over 2 weeks	Bilateral inferior parietal lobe	None
Liu et al. (36)	Randomized, sham-controlled crossover study	MCS PVS	5 2	Anoxic	4	1 session, 20 Hz rTMS (100% RMT) 1 session, sham rTMS with 48-h washout	Left M1	None
				Stroke/ICH	1			
				TBI	2			
Liu et al. (24)	Randomized, sham-controlled crossover study	MCSPVS	5 2	Anoxic	1	5 sessions, 20 Hz rTMS (100% RMT) 5 sessions, sham TMS with 1-week washout	Left M1	None
				Stroke/ICH	1			
				TBI	5			
Manganotti et al. (37)	Case series	MCSPVS	2 3	TBI	3	1 session, 20 Hz rTMS (120% RMT)	Left/right M1	None
				Stroke/ICH	2			
Naro et al. (31)	Case-Control with sham crossover in responders	UWS	10	Anoxic	10	1 session, 10 Hz rTMS (90% RMT) 1 session, sham TMS with 1-week washout*	DLPFC	None
Xia et al. (38)	Prospective single-blinded study	MCS UWS	5 11	Anoxia	5	20 sessions, 10 Hz rTMS (90% RMT)	Left DLPFC	None
				Stroke/ICH	9			
				TBI	2			

* *Naro et al. (31) conducted sham TMS only in the 3 patients demonstrating response to real rTMS*.

*CRS-R, Coma Recovery Scale-Revised; DLPFC, dorsolateral prefrontal cortex; DoC, Disorder of Consciousness; ICH, intracranial hemorrhage; M1, primary motor cortex; MCS, minimally conscious state; MO, machine output; PVS, persistent vegetative state; RMT, resting motor threshold; rTMS, repetitive TMS; TBI, traumatic brain injury; tDCS, transcranial direct current stimulation; TMS, transcranial magnetic stimulation; UWS, unresponsive wakefulness syndrome*.

**Table 2:** Overview of patient and protocol characteristics with number of responders.

**Characteristics**	**Number of responders**	**Total patients**	**Percent of patients with response**
**Diagnosis**
MCS	30	42	71.4%
PVS	11	48	22.9%
**Etiology**
Anoxic	16	47	34.0%
Stroke/ICH	10	17	58.8%
TBI	15	26	57.7%
**Time from injury to TMS**
Less than 3 months	6	10	60.0%
3 or more months	35	80	43.8%
**Number of sessions**
1	5	22	22.7%
More than 1 and <10	5	11	45.5%
10 or more	31	57	54.4%
**Stimulation site**
M1	7	23	30.4%
Non-M1	34	67	50.7%
**Stimulation frequency**
5 Hz	1	1	100%
10 Hz	14	28	50.0%
20 Hz	26	61	42.6%
**Stimulation intensity**
80% RMT or less	21	40	52.5%
More than 80% RMT	20	50	40.0%

*CRS-R, Coma Recovery Scale-Revised; DoC, Disorder of Consciousness; ICH, Intracranial hemorrhage; M1, primary motor cortex; MCS, minimally conscious state; PVS, persistent vegetative state; RMT, Resting motor threshold; TBI, traumatic brain injury; TMS, Transcranial magnetic stimulation*.

**Table 3:** Percent of patients with response stratified by disorder of consciousness and etiology.

**Disorder of consciousness and etiology of patients**						
	**MCS**			**PVS**		
	**Number of responders**	**Total patients**	**Percent of patients with response**	**Number of responders**	**Total patients**	**Percent of patients with response**
Anoxic	11	16	68.8%	5	31	16.1%
Stroke/ICH	5	6	83.3%	5	11	45.5%
TBI	14	20	70.0%	1	6	16.7%
Total	30	42	71.4%	11	48	22.9%

*ICH, Intracranial hemorrhage; MCS, minimally conscious state; PVS, persistent vegetative state; TBI, traumatic brain injury*.

**Table 4:** Number of patients with response stratified by disorder of consciousness and domain of CRS-R subscore improvement.

	**CRS-R subscore domains**					
	**Auditory**	**Visual**	**Motor**	**Oromotor/verbal**	**Communication**	**Arousal**
MCS	14	19	6	8	4	9
PVS	4	2	8	0	1	0
Total	18	21	14	8	5	9

*CRS-R, Coma Recovery Scale-Revised; MCS, minimally conscious state; PVS, persistent vegetative state*.

### Quality Assessment, Risk of Bias, and Heterogeneity of Included Studies

Quality assessment and risk of bias were assessed for each study, except for three case reports (Supplementary Figure 1). Heterogeneity among studies was very low for all three meta-analyses, with *I*^2^ = 0% and non-significant *p*-values. The overall quality of the studies was very low due to risk of bias, imprecision, and likelihood of publication bias. Risk of bias was found to be serious primarily due to concerns of inadequate blinding between intervention and evaluation of CRS-R score for some studies, leading to concerns in the measurement of outcomes domain.

### Synthesis of IPD and Meta-Analysis Results

**Figure 2:**
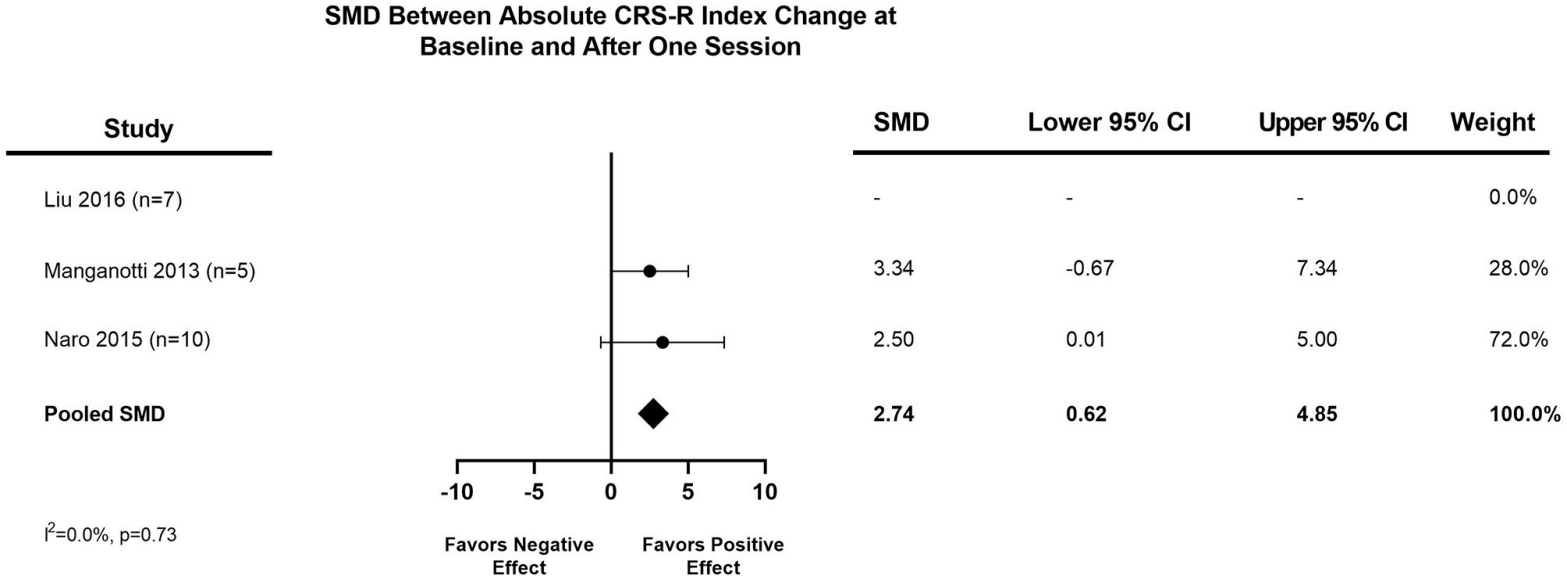
Meta-analysis of IPD: pooled mean absolute CRS-R index change after one session of TMS. A positive SMD (pooled mean absolute change toward the right of the figure) favors a positive effect for TMS, while a negative SMD (pooled mean absolute change toward the left of the figure) favors no effect or a negative effect for TMS. CI, Confidence Interval; CRS-R, Coma Recovery Scale-Revised; DoC, Disorder of Consciousness; SMD, standardized mean difference; TMS, Transcranial magnetic stimulation.

**Figure 3:**
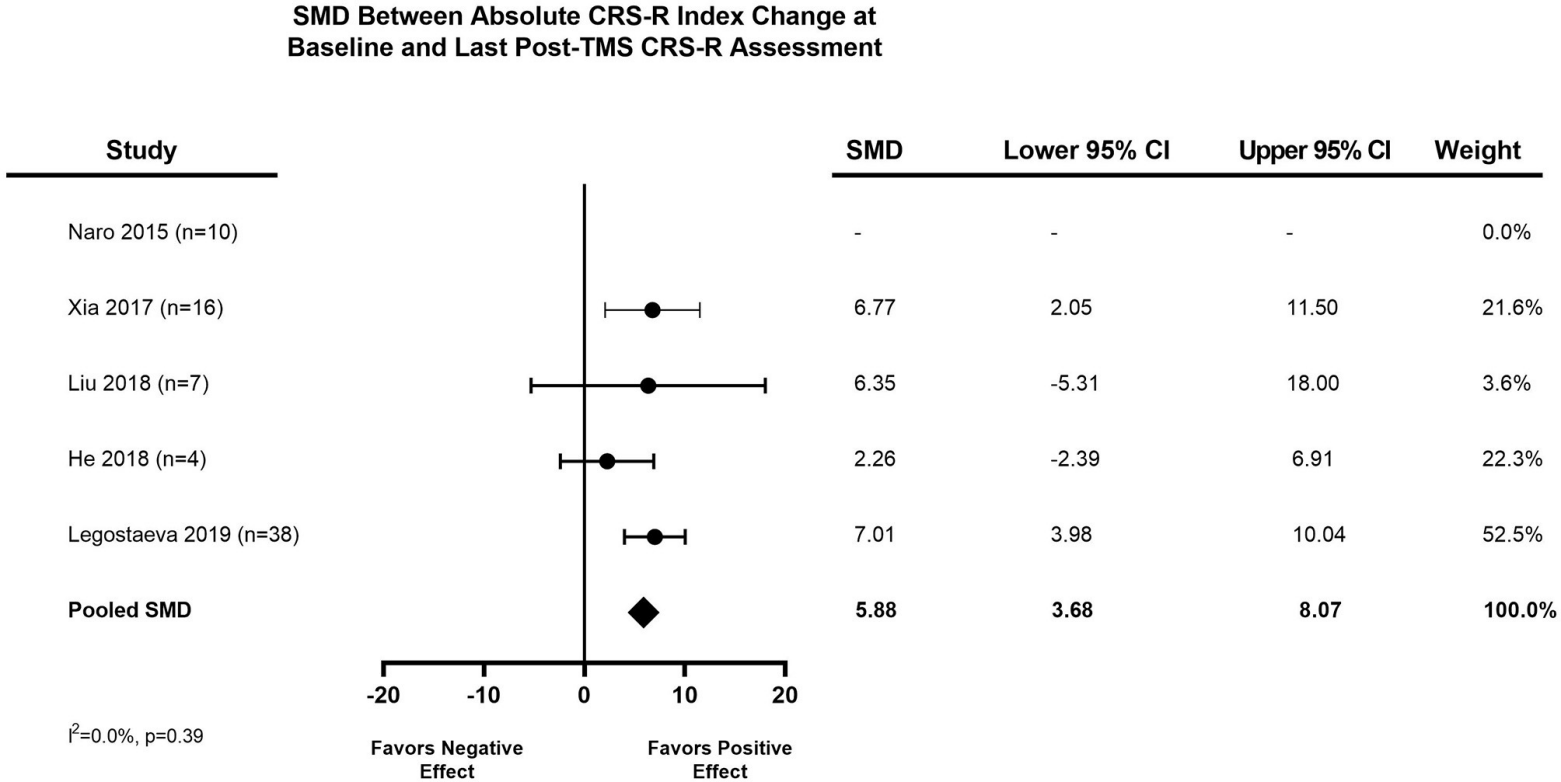
Meta-analysis of IPD: pooled mean absolute CRS-R index change from baseline to last Post-TMS CRS-R assessment. A positive SMD (pooled mean absolute change toward the right of the figure) favors a positive effect for TMS, while a negative SMD (pooled mean absolute change toward the left of the figure) favors no effect or a negative effect for TMS. CI, Confidence Interval; CRS-R, Coma Recovery Scale-Revised; DoC, Disorder of Consciousness; SMD, standardized mean difference; TMS, Transcranial magnetic stimulation.

**Figure 4:**
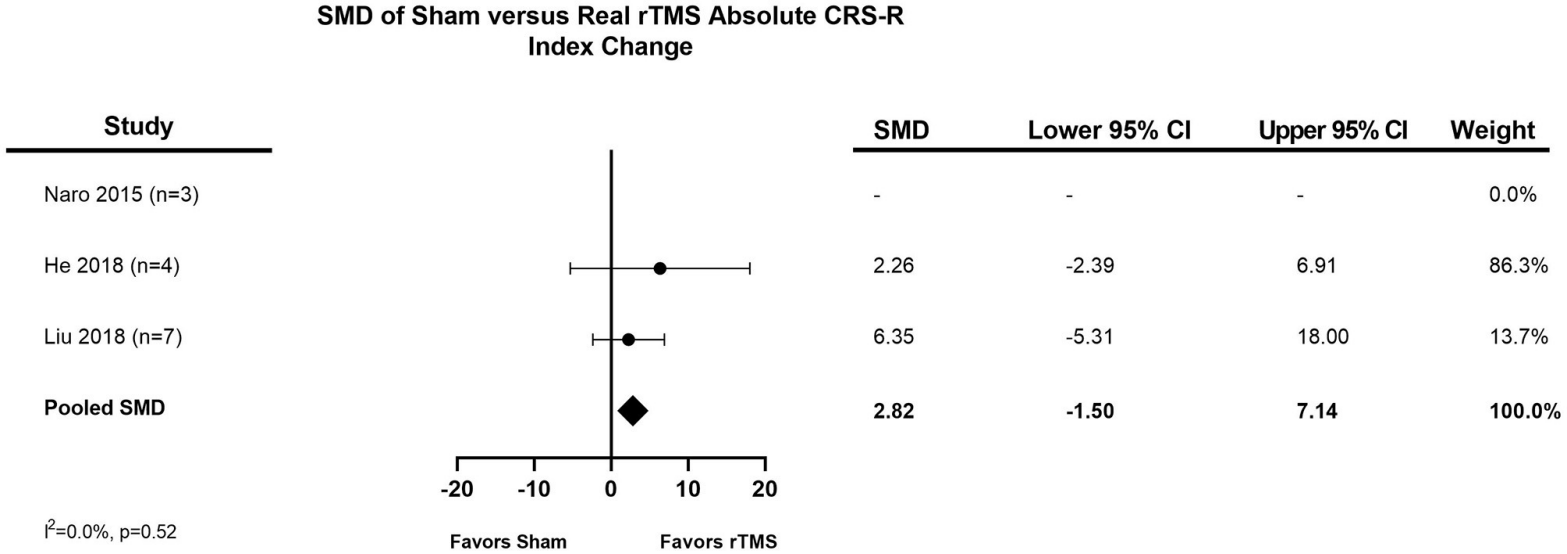
Meta-analysis of IPD: difference in pooled mean absolute CRS-R index change from baseline to last post-TMS CRS-R assessment for real rTMS vs. sham TMS. A positive SMD (pooled mean absolute change toward the right of the figure) favors a positive effect for TMS, while a negative SMD (pooled mean absolute change toward the left of the figure) favors sham TMS. CI, Confidence Interval; CRS-R, Coma Recovery Scale-Revised; DoC, Disorder of Consciousness; SMD, standardized mean difference; TMS, Transcranial magnetic stimulation.

The distribution of the mean pooled change in CRS-R Index after one session of TMS was 2.74 (95% CI, 0.62–4.85), as seen in Figure 2. The distribution of the pooled mean absolute CRS-R Index change from baseline to last post-TMS CRS-R assessment was 5.88 (95% CI, 3.68–8.07), demonstrated in Figure 3. Three studies provided control data for patients (24, 30, 31), two of which we were able to utilize to calculate a treatment effect (24, 30). The final meta-analysis assessing treatment effect of rTMS vs. sham TMS had a standardized mean difference of 2.82 (95% CI, −1.50 to 7.14) (Figure 4).

### Results of Additional Statistical Analysis: Paired *T*-Tests

Paired *t*-tests were performed to compare the mean pre-TMS CRS-R Index, or baseline score, to mean post-TMS CRS-R Index. CRS-R Index after one session of TMS was significantly higher than pre-TMS, with a mean difference of 1.813 (*p* = 0.0216, *N* = 23). CRS-R Index at last post-TMS assessment was significantly higher than pre-TMS, with a mean difference of 7.160 (*p* < 0.0001, *N* = 78), excluding patients with only one post-TMS score following one session of TMS. CRS-R Index at last post-TMS score reported for all patients was also significantly higher than pre-TMS, with a mean difference of 6.391 (*p* < 0.0001, *N* = 90).

### Results of Additional Statistical Analysis: Absolute Change in CRS-R Index Linear Regression Model

**Figure 5:**
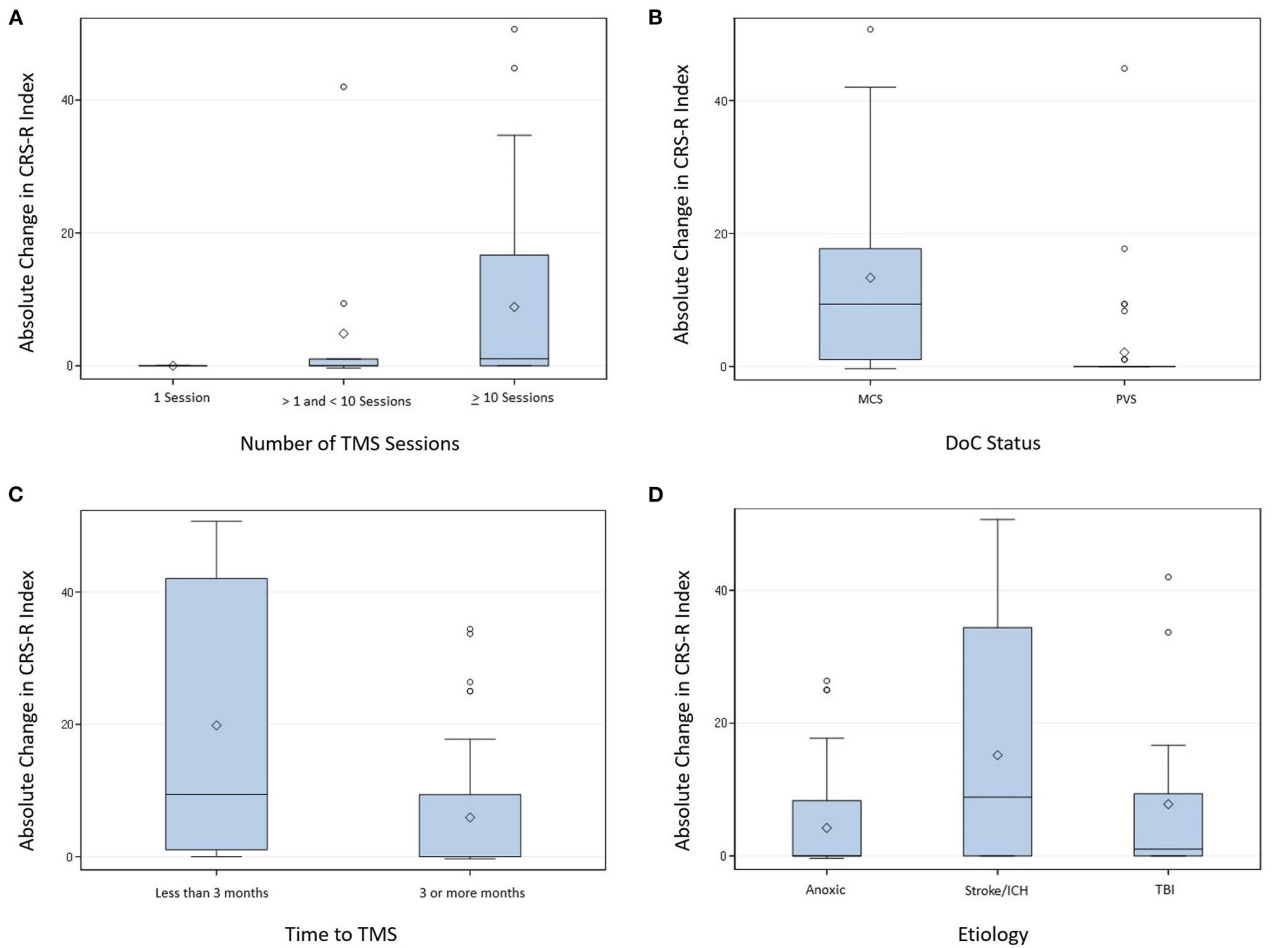
Distribution of mean absolute CRS-R index change from baseline to last post-TMS CRS-R assessment by number of TMS sessions **(A)**, DoC status **(B)**, time to TMS intervention **(C)**, and etiology **(D)**. CRS-R, Coma Recovery Scale-Revised; DoC, Disorder of Consciousness; ICH, Intracranial hemorrhage; MCS, minimally conscious state; PVS, persistent vegetative state; TBI, traumatic brain injury; TMS, Transcranial magnetic stimulation.

The following predictors were associated with significantly greater absolute CRS-R Index changes at the last post-TMS CRS-R assessment in the univariate analysis: receiving more than 10 sessions of TMS, having a diagnosis of MCS, receiving TMS within 3 months from inciting injury, and etiology of stroke/ICH. See Table 5 for predictors analyzed, their estimated difference in absolute CRS-R Index change between reference and evaluated levels, and the associated *p*-values and confidence intervals. Comparisons between significant predictors at the univariate level are also demonstrated in Figure 5 as boxplots showing the distribution of absolute CRS-R Index change.

**Table 5:** Results of univariate analysis: significance of predictors for absolute CRS-R index change.

**Predictor and reference level**	**Evaluated level**	**Estimated difference in absolute CRS-R index change between reference and evaluated level**	**95% CI lower limit**	**95% CI upper limit**	**Significance**
**Number of Sessions:** **>** 10*	**1 Session**	**−8.86**	**−16.72**	**−1.00**	**0.0303**
	More than 1 and <10	−4.00	−11.55	3.56	0.3029
Frequency: 20 Hz	5–10 Hz	1.70	−3.81	7.21	0.5475
Intensity: >80% RMT (High)	<80% RMT (Low)	3.87	−1.40	9.15	0.1539
Stimulation Site: Non-M1*	M1	−2.68	−10.33	4.97	0.4951
Etiology: Stroke/ICH*	**Anoxic**	**−10.95**	**−17.82**	**−4.08**	**0.0025**
	TBI	−7.38	−15.08	0.32	0.0644
**DoC Status: PVS**	**MCS**	**11.20**	**6.46**	**15.95**	** <0.0001**
Sex: Female*	Male	−1.45	−6.97	4.06	0.6071
**Time to TMS: 3 or more months**	**Less than 3 months**	**13.93**	**5.13**	**22.74**	**0.0027**

* *Significant levels are in bold. The significant levels in groups with three levels, “Number of Sessions: > 10” and “Etiology: Stroke/ICH,” were designated as reference levels so that direct comparisons could be made between their level and the other two levels evaluated. The levels “Stimulation Site: Non-M1” and “Sex: Female” were also designated as reference levels. In these cases, the reference level (which is equal to 0 for the purpose of calculating estimated differences in levels) is the absolute value of the estimated difference reported. For instance, > 10 sessions of TMS results in ~4.00 points of absolute change greater than more than 1 and <10 sessions*.

*CI, Confidence Interval; CRS-R, Coma Recovery Scale-Revised; DoC, Disorder of Consciousness; ICH, Intracranial hemorrhage; M1, primary motor cortex; MCS, minimally conscious state; PVS, persistent vegetative state; RMT, Resting motor threshold; TBI, traumatic brain injury; TMS, Transcranial magnetic stimulation*.

Significant univariate predictors were then tested for significance in the multivariate analysis using a linear regression model. In the final multivariate linear regression model, receiving more than 10 sessions of TMS, having a diagnosis of MCS, receiving TMS within 3 months from inciting injury, and etiology of stroke/ICH were all significantly associated with greater absolute CRS-R Index changes at the last post-TMS CRS-R assessment. See Table 6 for the estimated difference in absolute CRS-R Index change between reference and evaluated levels and the associated *p*-values and confidence intervals.

**Table 6:** Significant predictors of absolute CRS-R index change in multivariate linear regression model.

**Predictor and reference level**	**Evaluated level**	**Estimated difference in absolute CRS-R index change between reference and evaluated level**	**95% CI lower limit**	**95% CIupper limit**	**Significance**
**Number of Sessions:** **>** 10*	1 Session	−1.53	−8.55	5.50	0.6715
	**More than 1 and less than 10**	−9.63	−17.24	−2.03	0.0154
Etiology: Stroke/ICH*	**Anoxic**	−7.29	−13.70	−0.89	0.0288
	**TBI**	−7.96	−15.22	−0.70	0.035
**DoC Status: PVS**	**MCS**	10.78	5.90	15.66	<0.0001
**Time to TMS: 3 or more months**	**Less than 3 months**	13.61	4.17	23.06	0.0061

* *Significant levels are in bold. The significant levels in groups with three levels, “Number of Sessions: > 10” and “Etiology: Stroke/ICH,” were designated as reference levels so that direct comparisons could be made between their level and the other two levels evaluated. In these cases, the reference level (which is equal to 0 for the purpose of calculating estimated differences in levels) is the absolute value of the estimated difference reported. For instance, > 10 sessions of TMS results in approximately 9.63 points of absolute change greater than more than 1 and <10 sessions*.

*CI, Confidence Interval; CRS-R, Coma Recovery Scale-Revised; DoC, Disorder of Consciousness; ICH, Intracranial hemorrhage; MCS, minimally conscious state; PVS, persistent vegetative state; TBI, traumatic brain injury; TMS, Transcranial magnetic stimulation*.

## Discussion

In this meta-analysis, we found that TMS in patients with DoC favored a positive effect for TMS, as seen with positive standardized mean differences representing the pooled absolute change in CRS-R Index after one session of TMS and at the last post-TMS CRS-R assessment. Additionally, we found that patients in sham-controlled crossover studies demonstrated greater absolute changes in CRS-R Index scores following real TMS as compared to sham TMS. Patients with DoC who received TMS as an intervention also had significantly higher post-TMS CRS-R Index scores than pre-TMS CRS-R scores for all post-TMS time-points evaluated, though the greatest mean difference was noted in patients with more than one post-TMS assessment. Lastly, we created a linear regression model to evaluate the significance of patient- and protocol-specific factors on absolute change in CRS-R index. Patients were more likely to demonstrate a greater absolute change in CRS-R Index scores if they had a diagnosis of MCS, an etiology of stroke/ICH, if they received TMS within 3 months from inciting injury, or if they received at least 10 sessions of TMS.

### Evidence for TMS in DoC Patients

We found that patients with a diagnosis of MCS had greater absolute changes in CRS-R Index than patients with PVS (Figure 5), with an estimated absolute change in CRS-R Index of 10.78 points higher (Table 6). Out of 48 patients in PVS, only 11 responded to TMS (22.9%), as compared to response from 30 patients in MCS out of 42 (71.4%) (Table 3). However, it is important to consider the limitations of defining improvement above baseline as a true response in DoC patients. Table 4 shows the number of MCS and PVS patients demonstrating any response above baseline to TMS by domain of CRS-R subscore improvement. While MCS patients with response were most likely to improve in the visual or auditory domains, PVS patients were more likely to improve in the motor domain. Future studies are necessary to determine if there is a TMS protocol that may be more beneficial to PVS patients than the protocols utilized in the included studies.

Etiology may play an important role in patient response as well. Patients with stroke or ICH had significantly greater absolute changes in CRS-R Index scores compared to patients with anoxic or traumatic injuries, with an estimated 7.29 and 7.96 points greater absolute change, respectively (Table 6). Considering all significant patient-specific factors, MCS patients with stroke or ICH demonstrated response in 83.3% of patients, while PVS patients had a response rate of 45.5% (Table 3). Notably, the number of MCS and PVS patients with stroke or ICH are small. Further studies with larger patient cohorts and more uniform treatment protocols may help determine if this difference in response rates between etiologies could stratify which patients benefit from TMS.

### Proposing a TMS Protocol for DoC Patients

The number of TMS sessions patients received had a significant impact on absolute changes in CRS-R index, with patients receiving 10 or more sessions demonstrating the greatest absolute change (Figure 5). Just over half the patients receiving 10 or more TMS sessions demonstrated response to TMS, compared to improvement with one session or with more than one and <10 sessions of TMS, seen in 22.7 and 45.5% of patients, respectively (Table 3). This effect has been noted previously by Xia et al., who reported that only transient improvements in CRS-R scores were seen in MCS patients prior to completing 10 sessions of TMS (38). Future studies may consider utilizing 10 as the minimum number of sessions they require patients to complete for randomized, controlled trials of TMS. Comparing outcomes in patients receiving 10 sessions of sham vs. real TMS may further clarify the significance of this finding.

The time between inciting injury and TMS initiation was also a significant factor in determining changes in CRS-R Index. When TMS was initiated <3 months from injury, patients had significantly greater absolute CRS-R Index changes than those with TMS initiated after 3 months from injury (Figure 5). Data concerning the natural history of MCS and PVS prior to 3 months is limited, although MCS has been associated with improved prognosis compared to PVS (3). We suggest initiating TMS earlier than 3 months may have a positive impact on patient outcomes, though we acknowledge this finding is based on a very limited number of patients.

It is important to interpret these results cautiously. Time may be a confounding factor in this analysis, as patients with MCS and PVS can improve on their own with time. The results of our second meta-analysis showed a difference between the control and TMS groups in cross-over studies, suggesting absolute changes in CRS-R Index may be due to TMS rather than time. Although it is difficult to assess which patients are most likely to respond to TMS given the heterogeneity of treatment protocols and patient characteristics, it is important to note that TMS was performed without any reported adverse effects. From the 10 included studies, 8 discussed the safety of the TMS interventions utilized and explicitly reported that no patients experienced adverse effects (24, 31, 32, 34–38). The other two studies did not report any adverse effects, although no formal statement concerning safety of TMS was included in either (30, 33). Ethical concerns limit the potential for conducting research in this patient population (17). However, our results suggest that TMS in DoC patients is safe and has the potential to lead to improvement in CRS-R Index scores for some patients, possibly indicating the potential for improved outcomes.

All included studies utilized high frequency rTMS. Further studies are needed to characterize the dose-response to TMS, if indeed present in larger studies. Although stimulation site was not significant in our linear regression model, 50.7% of patients with TMS targeting non-M1 cortical areas demonstrated response to TMS, vs. those receiving TMS targeting M1 (30.4%). Future studies should evaluate which rTMS protocols may benefit patients most based on mechanism of rTMS at the proposed site of stimulation, possibly by assessing resting state functional connectivity of the DMN, which is known to have decreased internetwork connectivity possibly due to abnormally increased intranetwork connectivity (7, 9, 10).

Although not significant, high intensity protocols, or those with a stimulation intensity <80% RMT, demonstrated lower absolute changes in CRS-R Index scores than low intensity protocols (Table 5). Much of the existing data on what intensity to use is based on stimulating M1 and then applying the same parameters to other cortical regions. However, different neural networks and cytoarchitecture are present in cortical regions outside the motor cortex (39, 40). These findings may suggest that further studies on the impact of intensity in TMS protocols are required to characterize the most effective protocols for each cortical area.

### Limitations and Future Directions

In this meta-analysis, we identify patient and TMS protocol characteristics associated with the most improvement in CRS-R scores for PVS and MCS patients. Due to the lack of studies performing TMS in patients with DoC, our sample size is limited, with only 90 patients, of which 75 are included in the meta-analysis of IPD for absolute change in CRS-R index from baseline to the last reported score. It is also likely that some amount of selection bias occurred for studies that included IPD. We found IPD for more patients in PVS than MCS, with 48 and 42, respectively, and far more data for patients treated after 3 months from injury than prior to 3 months, with 80 and 10 patients, respectively. Detailed IPD were not available for all studies, and therefore we were unable to account for factors such as body mass index (BMI) or comorbid conditions.

Additionally, the TMS protocols analyzed are highly heterogenous, with differences at many levels of the TMS intervention. For example, studies utilized various stimulation sites, but relatively few protocols explored right-sided stimulation. For this reason, we were unable to explore the effect that side of stimulation had on change in CRS-R index. Despite these differences in protocols, our meta-analyses demonstrated very low heterogeneity. However, these results should be interpreted with caution due to the small sample size and low power of the included studies.

Although four of the studies included data for a control condition, the remaining six articles did not include such data and were lower in overall quality, ranging from case reports to prospective single-blinded studies. Additionally, control data was not available for all time points studied and therefore only three studies were included in the final meta-analysis of treatment effect. It is important to note that even in studies reporting control data, there are significant challenges present in achieving satisfactory sham TMS. Furthermore, patients may improve on their own, especially within the first 3 months of injury, making a true assessment of treatment effect difficult. Lastly, rTMS is a relatively new intervention for DoC, publication bias is likely present. This in addition to the lower quality of some studies limits the interpretation of these findings. Despite these limitations, we have provided what we believe to be the strongest available evidence for utilizing TMS in DoC patients and hope the synthesis of this data and comparisons of TMS protocols will aid investigators who are planning future studies. Larger, randomized-controlled trials are necessary to delineate any treatment effect that TMS may have in DoC patients and should compare TMS to amantadine, which is one of the few interventions currently suggested to improve DoC patient outcomes (3).

## Conclusions

We have provided a comprehensive analysis of the available data for TMS interventions in patients with DoC. There were no adverse effects reported in any studies, suggesting a trial of TMS may be considered in this patient population. We have described the aspects of TMS protocols associated with the most significant absolute change in CRS-R Index scores, in addition to some patient characteristics associated with a higher percent response. Although the number of studies utilizing TMS in DoC patients continually expands, not all institutions are able perform the large, randomized controlled trials necessary to improve the level of evidence for therapeutic TMS.

## Data Availability Statement

The original contributions presented in the study are included in the article/Supplementary Material, further inquiries can be directed to the corresponding author/s.

## Author Contributions

CO'N, TS, CG, and AC: study conception and design. CO'N, LS, AW, and SC: data acquisition and analysis. CO'N, LS, TS, AW, SC, CG, and AC: data interpretation, drafting and revising the manuscript. All authors contributed to the article and approved the submitted version.

## Author Disclaimer

The content is solely the responsibility of the authors and does not necessarily represent the official views of the National Institutes of Health.

## Conflict of Interest

The authors declare that the research was conducted in the absence of any commercial or financial relationships that could be construed as a potential conflict of interest.

## Publisher's Note

All claims expressed in this article are solely those of the authors and do not necessarily represent those of their affiliated organizations, or those of the publisher, the editors and the reviewers. Any product that may be evaluated in this article, or claim that may be made by its manufacturer, is not guaranteed or endorsed by the publisher.
