# Supplementary material for: Patient Outcomes in Disorders of Consciousness Following Transcranial Magnetic Stimulation: A Systematic Review and Meta-Analysis of Individual Patient Data
Source: Front Neurol. 2021 Aug 12;12:694970. doi: 10.3389/fneur.2021.694970 (PMC8407074; doi:10.3389/fneur.2021.694970)
Supplement: Supplementary file 1 [file Data_Sheet_1.docx]

Supplementary Material

# 1 Supplementary Figures and Tables

Table e-1. Detailed Characteristics of Included Studies

| **Study** | **Study Inclusion/Exclusion Criteria** | **Other Interventions** | **Medications** | **Length of Follow-up** | **Results at Follow-up** |
| --- | --- | --- | --- | --- | --- |
| Bai Y. et al., 2016 | - | Comprehensive brain injury rehabilitation program x 7 months before TMS | - | T1- immediately after last TMS session  T2- 30 days after start of TMS protocol | Improvement to MCS + (CRS-R of 14) |
| He F. et al., 2018 | Inclusion: no use of centrally acting drugs; no use of neuromuscular function blockers and no sedation within the prior 24 hours; periods of eye opening indicating a preserved sleep-wake cycle; a diagnosis of VS, MCS, or EMCS*  Exclusion: CI to rTMS, other severe systemic or neuro disease | - | - | T1- immediately after last TMS session  T2- 1 week after last TMS session | No significant effect on group level, but one patient did show clinical improvement. |
| Jang SH, et al., 2020 | - | Craniectomy with coiling of ruptured aneurysm, VP shunting with cranioplasty, comprehensive rehabilitative therapy concurrent with rTMS | Levodopa, amantadine, bromocriptine, zolpidem, baclofen | 2 months after last TMS session | Improvement to MCS (CRS-R of 13) |
| Legostaeva L. et al., 2019 | Exclusion: Non-stable clinical status (acute MI, DVT, PE, infections, sepsis, severe anemia), CI to MRI, epileptiform discharges on EEG, less than 1 year after TBI, less than 3 months of anoxia | 10 sessions physical therapy- 45-55 min each; Robotic verticalization x 10 sessions | - | 2 days after last TMS session | MCS patients- mean increase in total CRS-R score of 2.1  VS patients- no significant change |
| Lin Y. et al., 2019 | - | - | MCS1: gangliosides, ambroxol, imipenem, valsartan, captopril  UWS1: edaravone, mannitol, amlodipine, ampenem, vancomycin, reduced glutathione, ambroxol, famotidine | T1- immediately after last TMS session  T2- 4 weeks after last TMS session | Improvement in MCS patient |
| Liu P. et al., 2016 | Inclusion: no use of centrally acting drugs, no use of neuromuscular function blockers and no sedation within the prior 24 hours; periods of eye opening indicating a preserved sleep-wake cycle; diagnosis of VS or MCS  Exclusion: CI to rTMS, extracranial or intracranial artery disease, poor insonation of temporal bone windows | - | - | Immediately after TMS session | No significant change |
| Liu X. et al., 2018 | Inclusion: no use of drugs modifying cortical-excitability (beyond L-DOPA, baclofen and anti-epileptic drugs); no use of sedatives or neuromuscular blockers within 24 hours; no history of epilepsy, other severe neurological or systemic disease; no critical condition (respiratory or hemodynamic instability); no contraindication to TMS; open eyes; right-handed | - | - | 48 hours after last TMS session | No significant improvement overall, but one patient did show clinical improvement. |
| Manganotti P. et al., 2013 | Inclusion: absence of contraindications to TMS; stability of vital parameters; and >12 months since injury event | - | - | 6 hours after TMS session | No significant change overall with one patient showing improvement |
| Naro A. et al., 2015b | Inclusion: > 3 months after diagnosis of post-anoxic UWS; no other severe neurological or systemic diseases; no critical conditions; no cortical excitability modifying  drugs (L-DOPA, baclofen, and AEDs allowed); no history of epilepsy; no contraindication to TMS; no severe EEG abnormalities; no focal lesions in frontal lobes on MRI | - | - | T1- immediately after TMS session  T2- 60 minutes after TMS session | No significant changes were noted at a group level, but 3 patients had transient, clinical improvement. |
| Xia X. et al., 2017a | Inclusion: > 3 months after injury causing DoC; no improvement in consciousness; no other severe neurological or systemic diseases; no critical conditions; no history of epilepsy; no contraindication to TMS; and no focal lesion in the left DLPFC | Routine medication and rehabilitation course in the first month of their admission | All- Amantadine  MCS1, MCS4, PVS1-4, PVS7, PVS10-11 also received Baclofen | 10 days after last TMS session | 5/5 MCS and 4/11 UWS had significantly improved CRS-R scores. All had significantly improved CRS-R scores compared to the baseline (*p* = 0.007), though more notable in MCS (*p*=0.042) than UWS (*p*=0.066). |

* He F. et al. included a patient with a diagnosis of EMCS who had a baseline CRS-R score of 23 and was therefore not included in any analysis of IPD.

Abbreviations: AED= Antiepileptic drugs; CRS-R= Coma Recovery Scale-Revised; CI= contraindication; DLPFC= dorsolateral prefrontal cortex; DoC= Disorder of Consciousness; DVT= deep vein thrombosis; EEG= electroencephalogram; EMCS= emergence from MCS; ICH= intracranial hemorrhage; L-DOPA= Levodopa; M1= primary motor cortex; MCS= minimally conscious state; MI= myocardial infarction; MO= machine output; MRI= magnetic resonance imaging; PE= pulmonary embolism; PVS= persistent vegetative state; RMT= resting motor threshold; rTMS= repetitive TMS; TBI= traumatic brain injury; tDCS= transcranial direct current stimulation; TMS= transcranial magnetic stimulation; UWS= unresponsive wakefulness syndrome; VP= ventriculoperitoneal; VS= vegetative state

Table e-2. IPD by Study

| **Study** | **Patient Characteristics** | | | | | |
| --- | --- | --- | --- | --- | --- | --- |
|  | **Classification** | **Age & Sex** | **Etiology** | **Time from Injury to TMS (months)** | **CRS-R before TMS** | **CRS-R after TMS** |
| Bai Y. et al., 2016 | MCS1 | 47 F | S/ICH | 9 | 8 | 13; 14  (T1; T2) |
| He F. et al., 2018 | MCS1 | 14 M | Anoxia | 4 | 16 | 16;15  (T1; T2) |
|  | PVS1 | 49 F | TBI | 4 | 7 | 7 |
|  | PVS2 | 45 F | TBI | 28 | 7 | 7 |
|  | PVS3 | 58 M | S/ICH | 2 | 6 | 8 |
| Jang SH, et al., 2020 | PVS1 | 45 M | S/ICH | 5 | 4 | 13 |
| Legostaeva L. et al., 2019 | MCS1 | 67 F | Anoxia | - | 15 | 18 |
|  | MCS2 | 28 F | TBI | - | 15 | 20 |
|  | MCS3 | 56 F | Anoxia | - | 18 | 20 |
|  | MCS4 | 25 F | TBI | - | 7 | 9 |
|  | MCS5 | 24 F | TBI | - | 8 | 10 |
|  | MCS6 | 53 M | Anoxia | - | 9 | 12 |
|  | MCS7 | 44 M | TBI | - | 14 | 16 |
|  | MCS8 | 33 M | Anoxia | - | 12 | 13 |
|  | MCS9 | 48 M | Anoxia | - | 10 | 12 |
|  | MCS10 | 48 M | Anoxia | - | 9 | 10 |
|  | MCS11 | 23 M | TBI | - | 12 | 13 |
|  | MCS12 | 47 M | Anoxia | - | 10 | 10 |
|  | MCS13 | 18 M | TBI | - | 11 | 11 |
|  | MCS14 | 24 M | TBI | - | 13 | 13 |
|  | MCS15 | 32 F | Anoxia | - | 18 | 21 |
|  | MCS16 | 24 F | TBI | - | 20 | 22 |
|  | MCS17 | 31 F | Anoxia | - | 18 | 20 |
|  | MCS18 | 43 F | Anoxia | - | 13 | 19 |
|  | MCS19 | 32 M | TBI | - | 18 | 20 |
|  | MCS20 | 55 M | Anoxia | - | 15 | 18 |
|  | MCS21 | 20 M | TBI | - | 20 | 21 |
|  | MCS22 | 29 M | TBI | - | 20 | 22 |
|  | PVS1 | 22 F | Anoxia | - | 4 | 4 |
|  | PVS2 | 27 F | Anoxia | - | 4 | 4 |
|  | PVS3 | 31 F | Anoxia | - | 7 | 7 |
|  | PVS4 | 47 F | Anoxia | - | 5 | 5 |
|  | PVS5 | 19 F | TBI | - | 6 | 6 |
|  | PVS6 | 24 F | Anoxia | - | 7 | 7 |
|  | PVS7 | 47 F | Anoxia | - | 6 | 6 |
|  | PVS8 | 55 M | Anoxia | - | 4 | 4 |
|  | PVS9 | 21 M | Anoxia | - | 4 | 4 |
|  | PVS10 | 51 M | Anoxia | - | 5 | 5 |
|  | PVS11 | 22 M | Anoxia | - | 6 | 6 |
|  | PVS12 | 52 M | Anoxia | - | 5 | 5 |
|  | PVS13 | 47 M | Anoxia | - | 6 | 6 |
|  | PVS14 | 59 M | Anoxia | - | 6 | 6 |
|  | PVS15 | 31 M | Anoxia | - | 6 | 6 |
|  | PVS16 | 25 M | Anoxia | - | 6 | 6 |
| Lin Y. et al., 2019 | MCS1 | 31 M | S/ICH | 1 | 10 | 12; 19  (T1; T2) |
| Liu P. et al., 2016 | MCS1 | 62 M | TBI | 6 | 6 | 6 |
|  | MCS2 | 15 M | Anoxia | 4 | 9 | 9 |
|  | MCS3 | 42 M | S/ICH | 1 | 13 | 13 |
|  | MCS4 | 50 M | Anoxia | 2 | 12 | 12 |
|  | MCS5 | 63 M | TBI | 2 | 9 | 9 |
|  | PVS4 | 62 M | Anoxia | 4 | 7 | 7 |
|  | PVS5 | 30 F | Anoxia | 3 | 5 | 5 |
| Liu X. et al., 2018 | MCS1 | 60-65 | TBI | 6 | 6 | 7 |
|  | MCS2 | 10-15 | Anoxia | 4 | 16 | 16 |
|  | MCS3 | 40-45 | TBI | 1 | 15 | 23 |
|  | MCS4 | 60-65 | TBI | 2 | 6 | 7 |
|  | MCS5 | 50-55 | TBI | 2 | 13 | 13 |
|  | PVS1 | 46-50 | TBI | 5 | 7 | 7 |
|  | PVS2 | 56-60 | S/ICH | 2 | 6 | 7 |
| Manganotti P. et al., 2013 | MCS2 | 29 M | TBI | 94 | 10 | 11 |
|  | MCS3 | 38 M | TBI | 36 | 8 | 8 |
|  | PVS1 | 37 F | TBI | 34 | 7 | 8 |
|  | PVS2 | 67 M | S/ICH | 31 | 11 | 11 |
|  | PVS3 | 27 M | S/ICH | 12 | 2 | 2 |
| Naro A. et al., 2015b | UWS1 | F, 40 | Anoxia | 13 | 7 | 8; 7  (T1; T2) |
|  | UWS2 | F, 74 | Anoxia | 14 | 4 | 4; 4 |
|  | UWS3 | M, 71 | Anoxia | 14 | 3 | 3; 3 |
|  | UWS4 | M, 38 | Anoxia | 15 | 7 | 8; 7 |
|  | UWS5 | F, 55 | Anoxia | 13 | 6 | 6; 6 |
|  | UWS6 | M, 62 | Anoxia | 12 | 3 | 3; 3 |
|  | UWS7 | F, 32 | Anoxia | 4 | 7 | 8; 7 |
|  | UWS8 | F, 68 | Anoxia | 13 | 4 | 4; 4 |
|  | UWS9 | M, 70 | Anoxia | 12 | 3 | 3; 3 |
|  | UWS10 | F, 79 | Anoxia | 12 | 4 | 4; 4 |
| Xia X. et al., 2017a | MCS1 | M, 23 | TBI | 13 | 7 | 9 |
|  | MCS2 | F, 47 | S/ICH | 6 | 8 | 13 |
|  | MCS3 | F, 31 | Anoxia | 35 | 9 | 11 |
|  | MCS4 | M, 44 | S/ICH | 3 | 9 | 10 |
|  | MCS5 | M, 47 | S/ICH | 3 | 7 | 10 |
|  | UWS1 | M, 67 | S/ICH | 4 | 5 | 5 |
|  | UWS2 | F, 26 | S/ICH | 4 | 6 | 6 |
|  | UWS3 | M, 39 | S/ICH | 4 | 7 | 10 |
|  | UWS4 | M, 40 | S/ICH | 16 | 6 | 6 |
|  | UWS5 | M, 27 | S/ICH | 11 | 4 | 4 |
|  | UWS6 | M, 52 | S/ICH | 4 | 5 | 5 |
|  | UWS7 | M, 60 | Anoxia | 3 | 6 | 6 |
|  | UWS8 | M, 42 | S/ICH | 6 | 7 | 8 |
|  | UWS9 | M, 35 | Anoxia | 3 | 5 | 6 |
|  | UWS10 | F, 51 | Anoxia | 6 | 6 | 8 |
|  | UWS11 | F, 50 | Anoxia | 8 | 6 | 6 |

Abbreviations: CRS-R= Coma Recovery Scale-Revised; DLPFC= dorsolateral prefrontal cortex; DoC= Disorder of Consciousness; ICH= intracranial hemorrhage; M1= primary motor cortex; MCS= minimally conscious state; MO= machine output; PVS= persistent vegetative state; RMT= resting motor threshold; rTMS= repetitive TMS; S/ICH= Stroke/ICH; T1= First Post-TMS CRS-R Score; T2= Last Post-TMS CRS-R Score; TBI= traumatic brain injury; tDCS= transcranial direct current stimulation; TMS= transcranial magnetic stimulation; UWS= unresponsive wakefulness syndrome

Table e-3. Summary of Studies Included in Analyses

| **Analysis** | **Outcome Measure** | **Post-TMS Time Points Considered** | **Studies included** | **Number of Studies** | **Number of Patients** | **Results** |
| --- | --- | --- | --- | --- | --- | --- |
| **Meta-Analysis of IPD** | Absolute Change in CRS-R Index Measure | After one session | Liu P. et al., Manganotti et al., and Naro et al. | 3 | 22 | Figure 2 |
|  |  | Last post-TMS CRS-R Index recorded* | He et al., Legostaeva et al., Liu X. et al., Naro et al., and Xia X. et al. | 5 | 75 | Figure 3 |
| **Meta-Analysis of IPD- Sham versus Real rTMS** | Difference in Absolute Change in CRS-R Index Between Sham and Real rTMS | Last post-TMS CRS-R Index recorded* | He et al., Liu X. et al., and Naro et al. | 3 | 14 | Figure 4 |
| **T-test** | Mean Pre-TMS CRS-R Index versus Mean Post-TMS CRS-R Index | After one session | Bai et al., Liu P. et al., Manganotti et al., and Naro et al. | 4 | 23 | Mean difference: 1.813 (p= 0.0216)  Total Sessions**: 23; (1 per patient) |
|  |  | Last Post-TMS score reported* | Bai et al., He et al., Jang et al., Legostaeva et al., Lin et al., Liu X. et al., Naro et al., and Xia X. et al. | 8 | 78 | Mean difference: 7.160 (p< 0.0001)  Total Sessions: 920; (11.8 per patient) |
|  |  | Last Post-TMS score reported for all patients | Bai et al., He et al., Jang et al., Legostaeva et al., Lin et al., Liu P. et al., Liu X. et al., Manganotti et al., Naro et al., and Xia X. et al. | 10 | 90 | Mean difference: 6.391 (p< 0.0001)  Total Sessions: 932; (10.4 per patient) |
| **Linear Regression Model** | Absolute Change in CRS-R Index Measure | Last post-TMS CRS-R Index recorded* | Bai et al., He et al., Jang et al., Legostaeva et al., Lin et al., Liu X. et al., Naro et al., and Xia X. et al. | 8 | 78 | See Table 4 for univariate results and Table 5 for multivariate results |

* Excluding patients with only one post-TMS measurement after 1 session of rTMS

** Bai et al. is the only study in this group where a patient received more than 1 session of real TMS, but the patient had only received one session of TMS at the time-point analyzed for this group. Therefore, only 1 session is counted for the patient in Bai et al. when calculating the average sessions of TMS per patient in the analysis of this group.

Abbreviations: CRS-R= Coma Recovery Scale-Revised; IPD= individual patient data; rTMS= repetitive TMS; TMS= transcranial magnetic stimulation

**Figure e-1. GRADE and Risk of Bias Assessment**

* Number of events is defined as number of patients with post-TMS improvement in CRS-R score and treatment effect is absolute change in post-TMS CRS-R Index. The three case reports that were included in the individual patient data analysis were not included in this quality assessment due to assumed overall lower quality of evidence.

**Explanations**

a. Some studies were concerning for inadequate blinding between intervention and evaluation of CRS-R score, differences between patients in duration of intervention and length of follow-up, or did not report that analyses were specified prior to obtaining results

b. Confidence intervals for treatment effect overlapped with ranges for control conditions or reported no treatment effect

c. Overall number of studies is small and although there are negative studies reported in the literature, TMS as an intervention for DoC is relatively new and therefore likely susceptible to publication bias.

Abbreviations: CI= Confidence Interval; CRS-R= Coma Recovery Scale-Revised; DoC= Disorder of Consciousness; No= Number; rTMS= repetitive TMS; TMS= transcranial magnetic stimulation
